# Inflammatory mechanisms of mental illness: brain inflammatory response to interferon stimulation

**DOI:** 10.1192/bjo.2021.684

**Published:** 2021-06-18

**Authors:** James Herron, Jonathan Cavanagh

**Affiliations:** 1 University of Glasgow, NHS Greater Glasgow & Clyde; 2 University of Glasgow

## Abstract

**Aims:**

We hypothesise that peripheral IFN stimulation results in a brain inflammatory response via pathways of neuroimmune communication which in turn results in sickness-behaviour and depressive phenotype. We aim to determine if peripheral IFN stimulation results in brain inflammatory response including upregulation of inflammatory cytokines and chemokines.

**Background:**

There is increasing interest in the role of dysregulated immune function and inflammation in the pathogenesis of psychiatric disorders including mood disorders and dementias. Immune mechanisms offer a new approach to investigating mechanism in addition to offering hope for new avenues of treatment.

Interferon (IFN) therapy in humans is known to be associated with a significant risk of developing depression, both during therapy and increasing risk of relapse in the years following exposure, yet the mechanism remains unclear. IFN stimulation in animal models may offer insights into this phenomenon, in addition to furthering our understanding the role of immune mechanisms in the development of psychiatric phenotypes.

**Method:**

Mice (n. 42) were exposed to either IFN-alpha, IFN-gamma or vehicle control using either osmotic pump or intraperitoneal injection over the course of 7 days. Mice were scarificed, brains were dissected and RNA extracted. Inflammatory gene transcription within the brain was determined using real time quantitative polymerase chain reaction (RTqPCR). Absolute quantification was achieved using standard curves and reference gene. Statistical significance was determined using Mann-Whitney or ANOVA/Kruskal-Wallis depending on normality of data and number of groups.

**Result:**

IFNγ stimulation is associated with a significant brain upregulation of a number of inflammatory cytokines and chemokines including Il1β, Tnfα, Il10, Ifnγ, Ccl2, Ccl5, Ccl19, Cxcl10 and Ccr5. However, unexpectedly we did not find IFNα stimulation to associate with brain inflammatory transcriptional changes.

**Conclusion:**

This work demonstrates a brain inflammatory response to peripheral IFNγ stimulation. The inflammatory profile, including upregulated chemokines, suggests that recruitment of leukocytes across the blood brain barrier may be part of the immune response. Further experiments using existing tissues will explore if there are structural/cellular changes within the brain parenchyma. Further experiments within the group will seek to demonstrate if IFN treatment associates with sickness behaviour in order to determine if this is a clinically meaningful model. Suprisingly, we did not see similar changes in the IFNα treated group, which requires further investigation.

Funding: University of Glasgow, The Sackler Trust

